# Drivers of achiasmatic meiosis: sexual antagonism versus heteromorphy-dependent aneuploidy across sex-chromosome divergence

**DOI:** 10.1093/g3journal/jkaf217

**Published:** 2025-09-19

**Authors:** Andres Barboza, Heath Blackmon

**Affiliations:** Interdisciplinary Program in Genetics and Genomics, Texas A&M University, College Station, TX 77840, United States; Department of Biology, Texas A&M University, College Station, TX 77840, United States; Interdisciplinary Program in Genetics and Genomics, Texas A&M University, College Station, TX 77840, United States; Department of Biology, Texas A&M University, College Station, TX 77840, United States

**Keywords:** sex chromosomes, recombination, sexual antagonism, achiasmatic meiosis, aneuploidy, crossing over

## Abstract

Crossing over during meiosis ensures proper chromosome segregation and promotes genetic diversity. In species with chromosomal sex determination, recombination between sex chromosomes is often reduced or eliminated; yet, the evolutionary forces driving this shift remain debated. One extreme outcome, achiasmatic meiosis, typically completely halts recombination in the heterogametic sex. Here, we use a population genetic model to compare 2 leading hypotheses for the evolution of achiasmy: (1) selection to reduce recombination load from sexually antagonistic alleles and (2) selection to avoid aneuploidy driven by heteromorphic sex chromosomes. We analyze how mutations promoting achiasmy can invade autosomes, X chromosomes, or Y chromosomes under each selective regime. Our results reveal that the Y chromosome provides the most permissive context for invasion due to male-limited expression and selection. Moreover, we predict a shift in the primary selective forces across the trajectory of sex-chromosome divergence: sexually antagonistic selection is more likely to drive achiasmy in young, homomorphic sex chromosomes, whereas heteromorphy-dependent aneuploidy becomes the primary force in highly diverged, heteromorphic sex chromosomes. These results provide a unified framework for understanding transitions to achiasmy across diverse taxa.

## Introduction

**Fig. 1.**
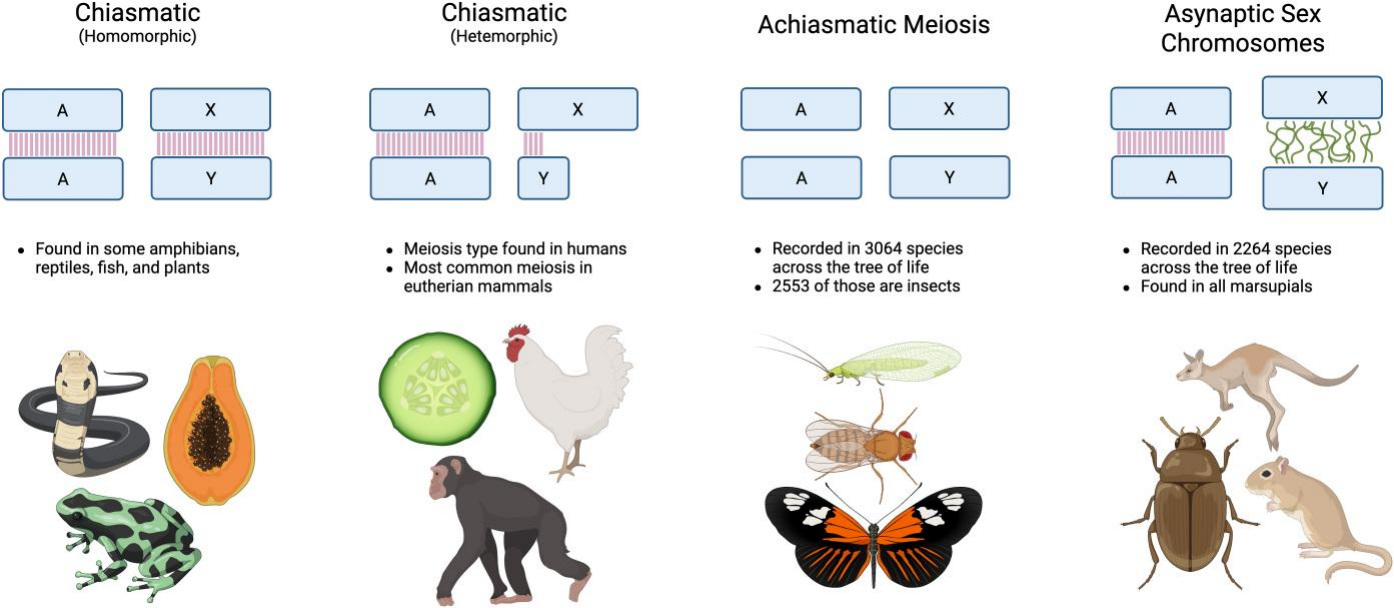
Meiotic mechanisms. Recombination during meiosis can vary greatly between organisms, but it can be broadly classified in the following 3 categories: Chiasmatic meiosis, where autosomal chromosomes recombine along their entire length and sex chromosomes recombine depending on their degree of divergence. Homomorphic sex chromosomes recombine along their entire length, while heteromorphic sex chromosomes recombine only in their PAR. Achiasmatic meiosis, where there is no recombination between autosomal or sex chromosomes in the heterogametic sex. Asynaptic sex chromosomes, where the autosomes recombine normally, but sex chromosomes are held at a distance with no opportunity for recombination. Areas with opportunity for recombination are shown as vertical lines. Curled lines show the protein structure known as the dense plate that separates asynaptic sex chromosomes during meiosis in marsupials (Smith 1978; Boyes 1980; Juan and Petitpierre 1991; Almeida et al. 2022). Created in BioRender. Barboza, A. (2025). https://BioRender.com/69kisxk.

Crossing over of chromosomes during meiosis is a crucial process that serves the dual purpose of allowing for recombination between homologous chromosomes and ensuring proper segregation of chromosomes into daughter cells. Both are typically essential for the fitness of an organism (but see: Mark Welch and Meselson 2000). Recombination, a consequence of crossing over, allows for the production of new haplotypes. On average, eukaryotes experience 1.5 cross over events, or chiasmata, per chromosome pair (Otto and Lenormand 2002; Otto and Payseur 2019). In mammals, lineages typically have either 1 crossover per chromosome or 1 crossover per chromosome arm (Dumont 2017). Recombination during gametogenesis accelerates adaptation and prevents accumulation of deleterious mutations (Muller 1932, 1964; Felsenstein 1974). However, this process can also disrupt associations between alleles at neighboring loci, which may be harmful if those alleles confer higher fitness when found together (ie coadapted gene complex) (Stebbins 1971; Charlesworth and Barton 1996; Neher and Shraiman 2009; Hipp et al. 2010). Despite the benefits of crossing over, many species with chromosomal sex determination exhibit limited recombination between sex chromosomes or the absence of recombination in the heterogametic sex (Bell 1981; Bachtrog et al. 2014; Tree of Sex Consortium 2014). The absence of recombination during gametogenesis is known as achiasmatic meiosis and is typically limited to the heterogametic sex. Limited research attributes changes in the expression of synaptonemal complex genes and specialized meiotic proteins as key features of achiasmatic meiosis (Thomas et al. 2005; John et al. 2016). Three broad categories of mechanisms lead to reductions in sex-chromosome recombination: modifications to the X and Y chromosomes that suppress recombination in a limited region (eg inversions), achiasmatic meiosis that suppresses all recombination in the heterogametic sex, and achiasmatic or asynaptic sex chromosomes that suppress recombination only in the sex chromosomes of the heterogametic sex (Fig. 1). However, despite extensive work on sex-chromosome evolution, we lack a clear understanding of what forces drive the complete cessation of recombination (achiasmatic meiosis) in the heterogametic sex, and how these forces vary across stages of sex-chromosome divergence.

### Hypotheses for the evolution of achiasmy

In our review of the literature, we find 5 hypotheses for the fixation of achiasmatic meiosis. In some cases, these hypotheses have focused largely on the evolution of heterochiasmy (differences in the recombination rate found in males and females) or inversions but can be naturally extended to the extreme case of achiasmy in the heterogametic sex. We will consider the first 2 hypotheses in our analysis.

#### Achiasmy driven by sexual antagonism

The Haldane–Huxley hypothesis is the first and still widely held hypothesis for the evolution of achiasmatic meiosis due to sexually antagonistic variation (Haldane 1922; Huxley 1928; Bull 1983: 18; Rice 1987). Intralocus sexual antagonism arises when a locus in a population harbors 2 alleles, one of which enhances fitness in males, while the other enhances fitness in females (Stewart et al. 2010). Sexually antagonistic loci in the recombining portion of the sex chromosome lead to recombination load (reduction in fitness due to recombination). This recombination load results in indirect selection on recombination rates, favoring genotypes with lower recombination and thus may favor the evolution of achiasmatic meiosis.

#### Heteromorphy-dependent aneuploidy

Another explanation for the fixation of achiasmy is heteromorphy-dependent rates of sex-chromosome aneuploidy (Blackmon and Demuth 2015; Blackmon and Brandvain 2017). This process, described as the fragile Y hypothesis, posits that aneuploidy rates of sex chromosomes are negatively correlated with the size of the pseudoautosomal region (PAR) of the sex chromosomes. This hypothesis is consistent with data on autosomal aneuploidy rates in humans, the parental source of sex-chromosome aneuploids, and the frequency of sex-chromosome aneuploids in domestic animals (Warburton and Kinney 1996; Hall et al. 2006; Raudsepp et al. 2012; McCoy et al. 2015). Achiasmatic meiosis allows for the reliable pairing of a sex chromosome in the absence of a PAR. Therefore, achiasmy causing mutations (hereafter referred to simply as achiasmy mutations) would benefit from producing a higher amount of viable gametes. The magnitude of this benefit becomes proportional to the rate of sex-chromosome aneuploidy of chiasmatic individuals.

#### Drift

Achiasmy mutations could have fitness effects that are effectively neutral and are fixed through drift. To our knowledge, this has yet to be explored. However, discussions of the way that inversions can become fixed via drift can be extended to the evolution of achiasmy. It is clear from theoretical work that neutral processes can allow inversions to become fixed via genetic drift (Charlesworth et al. 1987; Navarro and Barton 2003), and a number of empirical studies have suggested that many inversions are neutral or nearly so (Ledyard Stebbins 1975; Kuvangkadilok et al. 2003; Aradottir and Angus 2004; Kirkpatrick 2010). However, many empirical examples of inversions are not neutral, and this explanation alone seems unlikely to explain the presence of achiasmy across the Tree of Life nor its bias in occurring in the heterogametic sex (Satomura et al. 2019).

#### Differences in haploid selection

Heterochiasmy may be positively selected during the haploid phase of both sexes if linkage disequilibrium exists between the given loci (Lenormand 2003). The capacity for haploid selection in vertebrate females is limited, suggesting that if haploid selection drives heterochiasmy, it would primarily affect males (Lenormand and Dutheil 2005). If recombination suppression mutations arise, they will be positively selected in the sex where recombination is most detrimental due to intense haploid selection, potentially leading to achiasmy if the selection is strong and persistent. This hypothesis is most relevant to organisms with a long haploid phase (eg nonseed-bearing plants and fungi).

#### Complex epistasis

Differences in *cis*-epistasis and *trans*-epistasis between the sexes in the diploid phase could also lead to heterochiasmy. In this hypothesis, males and females may exhibit differences in the levels of selection due to the different combinations of genes favoring heterochiasmy (Lenormand 2003). If the difference in the recombination load between sexes is very large, selection could push recombination to 0 in 1 sex. For instance, if in the heterogametic sex, negative *cis*-epistasis overwhelmingly outweighs *trans*-epistasis, selection might strongly favor suppression of recombination entirely, resulting in achiasmy.

### Variation in genomic regions

When studying the evolution of achiasmy, it is important to account for the genomic location that holds this new mutation, as not all regions of the genome are equally suitable for a given gene. By “suitable,” we mean that certain genomic regions experience unique patterns of selection or transmission probabilities that can facilitate, or hinder, the fixation of specific mutations, such as those that cause achiasmy. Specifically, autosomes, X chromosomes, and Y chromosomes each exhibit different inheritance patterns, ploidy, and residence time in males and females. Autosomes are present in 2 copies in both females and males and are inherited equally from both mothers and fathers. In contrast, X chromosomes are diploid in females and haploid in males to the extent that the Y chromosome has diverged from the X chromosome. For instance, the human X chromosome has 866 coding genes, and only 18 of these are also present on the Y chromosome (Rosser et al. 2009; Bellott et al. 2014; Frankish et al. 2019). Thus, 97.3% of X chromosome genes are expressed in a haploid state in males. On the other hand, Y chromosomes are haploid, found only in males, and passed down exclusively through the paternal line. Due to these differences in the distribution among the sexes, chromosomes also differ in their effective population size with X chromosomes having an effective population size that is only 75% that of autosomes. Even more extreme, Y chromosomes have an effective population size that is just 25% of that of autosomes. Finally, while autosomes experience selection in males and females at equal rates, X chromosomes experience selection in females two-thirds of the time and only experience selection in males one-third of the time, and Y chromosomes experience selection only in males. However, X chromosomes are exposed to selection in males as they are hemizygous. This translates in a major impact into the evolutionary dynamics of an achiasmatic mutation and the balance between selection and drift, especially in the cases of sex-specific selection.

Work in retrogenes has illustrated several clear patterns that suggest that sex chromosomes can be a particularly advantageous or disadvantageous region for a gene, depending on its function. For instance, analyses of mosquito, silkworm, and kelp genomes have revealed that retained retrogenes are biased toward both originating from and moving to sex chromosomes (Toups and Hahn 2010; Wang et al. 2012; Lipinska et al. 2017). Similarly, genes involved in certain processes are enriched on sex chromosomes (Khil et al. 2004; Vallender and Lahn 2004). This is perhaps most striking with regard to genes involved in spermatogenesis, where we see the Y chromosome being enriched for such genes (Subrini and Turner 2021). This process of specialization is paralleled in the loss of nonmale-specific function of the Y chromosome (Ellis and Affara 2006). Such patterns could extend to mutations that cause sex-specific alterations to meiosis.

In this manuscript, we use a population genetic model to answer 2 fundamental questions concerning mutations that cause achiasmatic meiosis: (1) are achiasmatic mutations favored more strongly on specific chromosomes (autosome, X, Y), and (2) do sexual antagonism and heteromorphy-dependent aneuploidy make different predictions about the fixation of achiasmatic meiosis mutations?

## Methods

**Fig. 2.**
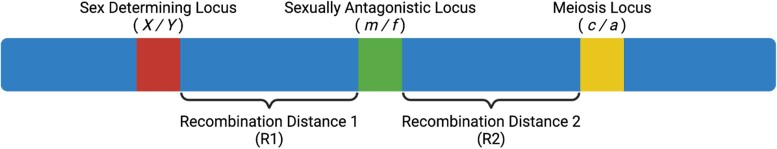
Genome model. Our model can represent a genome with 3 loci separated by an arbitrary distance ranging from 0 (complete linkage) to 0.5 (unlinked loci).

We constructed a model of a diploid organism with discrete, nonoverlapping generations and 3 biallelic loci (Fig. 2). The sex-determining locus (SDL) has alleles X and Y, with XX individuals being female and XY individuals being male. At the sexually antagonistic locus (SAL), allele *m* is male-beneficial, while allele *f* is female-beneficial, and the dominance factor *h* applies to allele *m*. The SAL is separated from the SDL by the recombination distance R1. We evaluate values of 0.1 and 0.3 for R1 representing tight and loose linkage between the SDL and SAL. The meiosis locus (ML) has 2 alleles, *c* for chiasmatic meiosis and *a* for achiasmatic meiosis. We do not allow for reversion mutations at the ML as there is little evidence that species that have achiasmatic meiosis are able to regain meiotic meiosis (but see: Wei and Bachtrog 2019). The ML is separated from the SAL by the recombination distance R2. We set R2 to 0.1 to represent the locus being on the sex chromosomes and a value of 0.5 to represent the locus being on an autosome. Future work evaluating whether the degree of linkage between the ML and SAL has an impact on the fate of meiotic mutations remains an area open for exploration.

We employ a symmetrical fitness function at the SAL where the sex of an individual and their genotype determine fitness, with *s* denoting the selection coefficient at this locus. Our model allows for chiasmatic meiosis to be associated with lower segregation fidelity (ie high aneuploidy rates). This aneuploidy load can be relieved in our model by switching to achiasmatic meiosis. The fitness cost of chiasmatic meiosis in this case is directly linked to the aneuploidy rate, denoted as *k* in the model.

Achiasmatic meiosis stops recombination in the heterogametic sex. The achiasmy allele *a* works to replicate this behavior, expressing in a dominant, sex-specific way to stop all recombination in the heterogametic sex. The homogametic sex remains unaffected and recombines normally. The cost associated with achiasmatic meiosis is the accumulation of deleterious mutations caused by Muller's ratchet. Autosomes and X chromosomes still recombine in the homogametic sex, so the cost is concentrated in the Y chromosome. Mutations are random in both frequency and effect. While some methods can account for the impact of a distribution of deleterious effects under Muller's ratchet (Butcher 1995; Schultz and Lynch 1997; Gordo and Charlesworth 2001), they would add unnecessary complexity to the model with at most marginal gains in biological accuracy (Loewe 2006). Additionally, previous work has shown that the rate of Muller's ratchet approximates a constant when considered as the rate at which the fittest class is lost, at which point it is described primarily as a function of the population size and the genomic mutation rate (Haigh 1978; Stephan et al. 1993; Gordo and Charlesworth 2000; Etheridge et al. 2007; Bräutigam and Smerlak 2022). In a deterministic model with an infinite population, only the mutation rate needs to be considered. This informs our model where the rate of mutational load is modulated by the genomic mutation rate (*μ*) and the number of coding sites now exposed to Muller's ratchet (*L*). The mutation rate is maintained at a biologically relevant constant (*μ* = 10^−9^), as determined by previous studies (Wielgoss et al. 2011; Scally 2016). In this model, mutations compound their effects multiplicatively, resulting in an exponential decline in fitness. The effect of each mutation was implemented as a constant (*δ =* 0.03) taken as the average value of the distribution of fitness effects of new mutations in coding sites (Boyko et al. 2008). Equation (1) illustrates how the mutational load for the Y chromosome is calculated, which allows us to explore parameter space across different levels of mutational burden as a function of the number of coding sites transitioning to achiasmy:


(1) $$\mathrm{Mutational}\;\mathrm{Load}=(1-\delta {)}^{Ut},$$

with *U = μL* and *t* being the time in generations. Table 1 demonstrates how components of fitness are combined multiplicatively to generate the final fitness of each genotype.

**Table 1.** Fitness function for the model's genotypes.

SAL locus	ML locus	Male	Female
*mm*	*ac/aa*	(1 + *s*)(1−δ)^Ut^	1/(1 + *s*)
*mf*	*ac/aa*	(1 + *hs*)(1−δ)^Ut^	1/(1 + *hs*)
*ff*	*ac/aa*	(1−δ)^Ut^	1
*mm*	*cc*	(1 + *s*)(1−*k*)	1/(1 + *s*)
*mf*	*cc*	(1 + *hs*)(1−*k*)	1/(1 + *hs*)
*ff*	*cc*	1−*k*	1

Each row represents a genotype, where the first 2 columns identify the genotype at each locus and the last 2 columns indicate the fitness of the phenotype for each sex. Note that *k* is set to zero unless the heteromorphy-dependent model is being analyzed and vice versa. At the SAL, allele *m* is male-beneficial, while allele *f* is female-beneficial, and the dominance factor *h* applies to allele *m*. The meiosis locus has 2 alleles: *c* for chiasmatic meiosis and *a* for achiasmatic meiosis.

We developed a system of difference equations that tracks the frequency of gametes in a population across generations under this model (Supplementary Material 1). Gametes are expressed with their genotypes, denoted with either an X or a Y for the SDL. The other 2 loci are denoted with subscripts: the SAL with subscript *m* or *f* and the ML with subscript *c* or *a*. We used a superscript to denote the gamete as an egg (♀) or a sperm (♂). There are 4 possible types of eggs (*X^♀^_mc_*, *X^♀^_ma_*, *X^♀^_fc_*, and *X^♀^_fa_*) and 8 possible types of sperm (*X*^♂^*_mc_*, *X*^♂^*_ma_*, *X*^♂^*_fc_*, *X*^♂^*_fa_*, *Y*^♂^*_mc_*, *Y*^♂^*_ma_*, *Y*^♂^*_fc_*, and *Y*^♂^*_fa_*). Mating in the population is random. The frequency of a gamete is the weighted sum of the frequencies of the genotypes that can produce said gamete and the fitness of those genotypes measured in adults.

We evaluated achiasmy mutations on Y and X chromosomes separately and on autosomes by increasing the recombination distance R2 to 0.5. The achiasmatic allele mutation started with a frequency of 0.0001 in the population, adjusted to account for the differences in the effective population size of each chromosome. This allows us to observe the behavior of this allele soon after its origin. For each of these mutation locations, we evaluated 200 measures of the degree of sex-chromosome divergence, specifically the number of sites that would transition to achiasmy over a range of 0 (highly diverged sex chromosomes) to 5 Mb (homomorphic sex chromosomes) on 25 Kb increments of *L*. The value of 5 Mb was chosen to reflect the approximate number of protein-coding sites in the ancestral mammalian sex chromosome prior to XY divergence. This value was taken from the current number of protein-coding sites on the human X chromosome. We acknowledge that mammals have not, to our knowledge, evolved achiasmatic meiosis. However, these assumptions allow us to ensure that our model is grounded in an area of parameter space that is sampled by empirical genomes. Finally, an additional concern is that we do not model autosome general fitness loci (eg any cost from Muller's ratchet is limited to the Y). We believe that this is an appropriate assumption because previous work has shown that even intermittent or occasional recombination is sufficient to halt the impact of Muller's ratchet (Perrin 2009; Rodrigues et al. 2018).

For each of these 600 combinations, we evaluated the fate of achiasmy mutations in the presence of sexual antagonism, evaluating 200 selection coefficients ranging from 0 to 0.5. In these analyses, we assume no aneuploidy in either achiasmatic or chiasmatic individuals (*k =* 0), and the sexually antagonistic alleles start at equal frequencies. For each of these parameter pairings, we evaluated the impact of dominance factors of 0, 0.5, and 1.

Next, we evaluated the model of heteromorphy-dependent aneuploidy rates with 200 measures of sex-chromosome aneuploidy rates ranging from 0.0 to 0.05, and no sexual antagonism (*s =* 0). These aneuploidy rates only impact males that are undergoing chiasmatic meiosis. Females or individuals with achiasmatic meiosis experience zero aneuploidy. This resulted in a final collection of 480,000 distinct conditions to be analyzed. For each condition, we iterated the difference equations across 1,000 generations, or until the achiasmatic mutation reached a frequency of 0.99.

## Results

### Impact of sexual antagonism

**Fig. 3.**
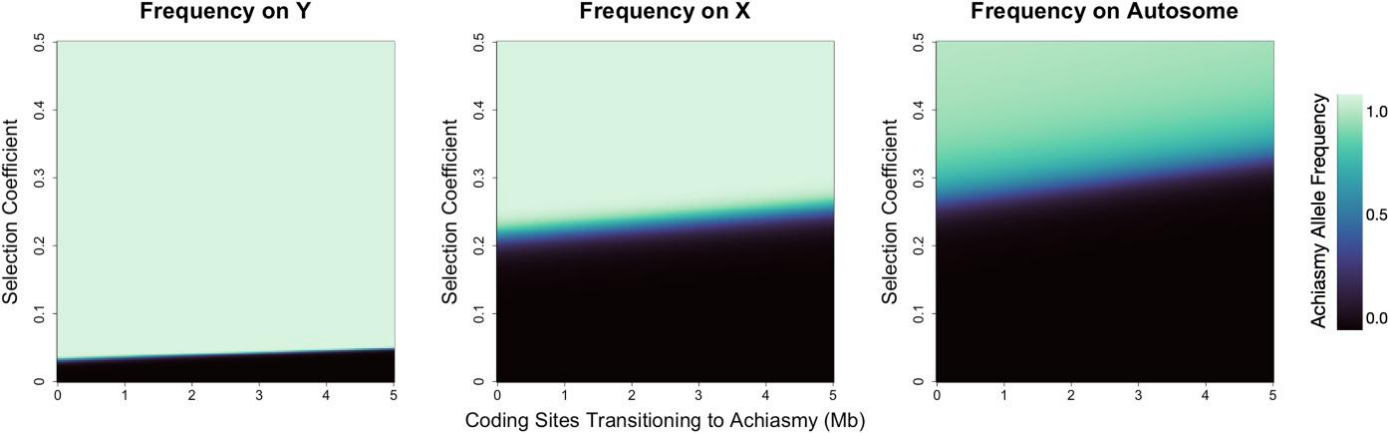
Sexual antagonism as the selective pressure on the ability of achiasmy mutations to fix. In each plot, the horizontal axis represents the size of the recombining region of the Y chromosome. It serves as an equivalent measurement for the degree of mutational load. High values on this axis represent homomorphic sex-chromosome pairs, leading to many genes shifting to a nonrecombining state on the Y chromosome. The vertical axis represents the selection coefficients explored. Colors in each plot represent the final frequency of the achiasmy mutation.

When sexual antagonism was the selective force, achiasmatic mutations fixed most readily on the Y chromosome, followed by the X chromosome, and then autosomes (Fig. 3). Y chromosomes have a higher tendency to fix these mutations across the studied parameter space and do so with faster dynamics, reaching equilibrium 4 times faster than when the mutation is on the X chromosome and 18 times faster than when it is on an autosome under the same parameters (Supplementary Fig. 1). The striking difference between the Y chromosome relative to the X chromosome and the autosomes is likely a function of both linkage with the SAL and sex-specific selection. Specifically, when a mutation is on a sex chromosome, it can be physically linked to an allele at the SAL that is beneficial to the sex most frequently carrying that chromosome (eg an X chromosome carrying the achiasmy mutation and the female benefit allele or a Y chromosome carrying the achiasmy mutation and the male benefit allele). This benefit is compounded in the case of the Y chromosome, where it is always present in males and thus experiences a single selection regime. Our evaluation of dominance factors shows a pattern where, as the dominance factor of the male benefit allele increases, the parameter space where the mutation fixes increases (Supplementary Figs. 3 and 4). This pattern is similar to that observed when studying changes in recombination distances in R1, where higher values increased the parameter space where the mutations are fixed (Supplementary Fig. 5). These patterns are largely driven by the fact that when the dominant allele benefits the heterogametic sex, sexual antagonism can be fully resolved with both sexes achieving maximum fitness.

### Impact of heteromorphy-dependent aneuploidy

**Fig. 4.**
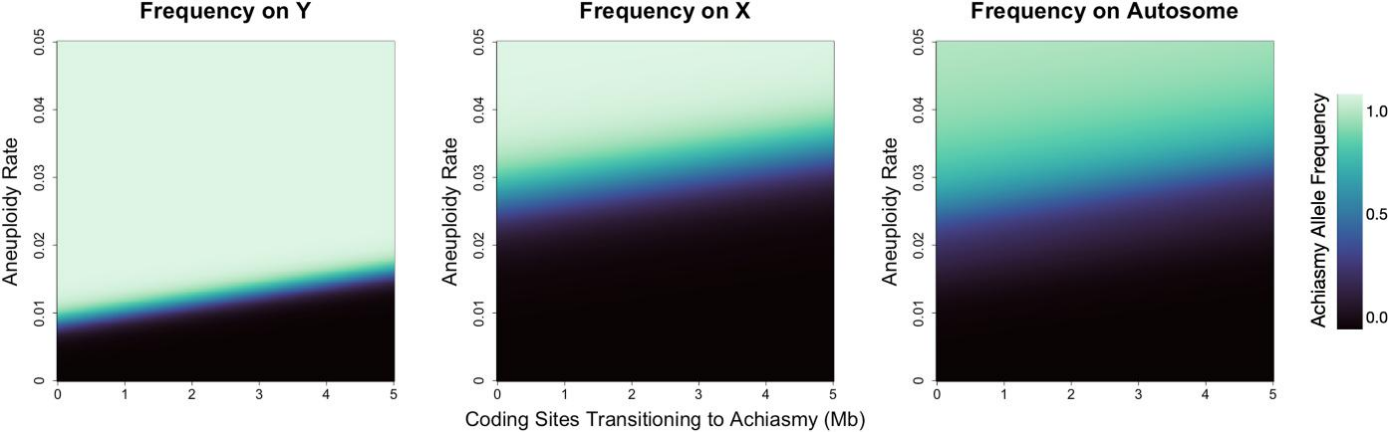
Heteromorphy-dependent aneuploidy as the selective pressure on the ability of achiasmy mutations to fix. In each plot, the horizontal axis represents the size of the recombining region of the Y chromosome. It serves as an equivalent measurement for the degree of mutational load. High values on this axis represent homomorphic sex-chromosome pairs, leading to many genes shifting to a nonrecombining state on the Y chromosome. The vertical axis represents the sex-chromosome aneuploidy rates explored. Colors in each plot represent the final frequency of the achiasmy mutation.

When heteromorphy-dependent aneuploidy was the selective force in the model (Fig. 4), the area of parameter space where the mutation was fixed in the population followed a similar pattern as observed in the previous section, with the Y chromosome most readily fixing this mutation followed by the X chromosome and then autosomes. However, the parameter space permitting invasion of this mutation is broader on autosomes than on the X chromosome, likely because autosomes reside in males half the time, whereas X chromosomes do so only one–third of the time, yielding different responses to sex–specific selection. The dynamics followed a similar pattern, with the mutation reaching equilibrium on the Y 3 times faster than when the mutation is on the X chromosome and 13 times faster than when it is on an autosome under the same parameters (Supplementary Fig. 2). The deviation from the sexual antagonism model is largely driven by the fact that no linkage with any specific locus is necessary; rather, differences among chromosomes are likely due to residence time in males. The differences observed across these 2 models suggest that the genomic distribution of achiasmy mutations may be more uniform if the driving force is aneuploidy reduction. Though we present our work in terms of a male heterogametic system, the expected dynamics would be identical in a ZZ/ZW system.

## Discussion

Our results suggest that both sexual antagonism and heteromorphy-dependent aneuploidy rates may drive the fixation of achiasmy. Additionally, our model makes very similar predictions about the genomic region where these mutations are expected to be found. Future work identifying the gene(s) responsible for transitions to achiasmy may help us to better understand the most probable forces underlying the evolution of achiasmy. While causative genes for achiasmy have not been identified, it is likely that there may be genes that are currently annotated as involved in meiosis or spermatogenesis. Concordant with our results, there is evidence of a concentration of genes involved in meiosis and spermatogenesis on the human and mouse Y chromosome (Pagani et al. 2002; Subrini and Turner 2021). In order to corroborate this trend, we performed a Gene Ontology term enrichment analysis on Y chromosomes. In the human Y, “spermatogenesis” and “gonadal mesoderm development” were significantly overrepresented. In *Drosophila melanogaster*'s Y chromosome, the term “sperm DNA condensation” was significantly overrepresented (Supplementary Methods). This underscores the enrichment of genes involved in male gametogenesis in the Y chromosome.

**Fig. 5.**
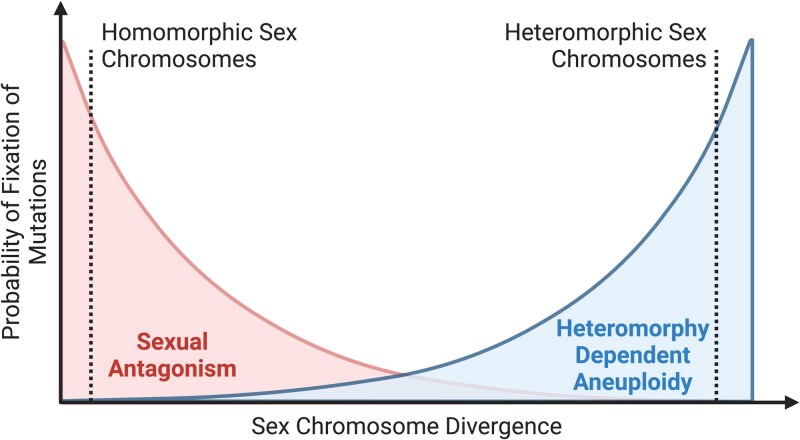
Probability of fixation of achiasmatic mutations across the divergence of sex chromosomes. This figure illustrates our hypothesis that sexual antagonism is more likely to drive the early fixation of achiasmy, while heteromorphy-dependent aneuploidy is more likely to drive transitions to achiasmy in older sex chromosomes.

When we synthesize our results with the canonical model of sex-chromosome evolution, we also discover that our model suggests that the most probable force driving the fixation of achiasmy mutations likely changes as sex chromosomes diverge. When sex chromosomes are largely homomorphic, the area available for recombination is high (ie low probability of aneuploidy), and the number of genes loosely linked to the SDR is high (high probability of sexual antagonism). Furthermore, with largely homomorphic sex chromosomes, we would expect mutational load to be relatively high since many genes would become nonrecombining in the event of an achiasmatic mutation. Thus, homomorphic sex chromosomes would fall at the top of plots in Fig. 3 and at the bottom of plots in Fig. 4. In contrast, highly diverged sex chromosomes like those found in most mammals would show an opposite pattern where the opportunity for sexual antagonism is reduced since fewer genes are loosely linked to the SDR, but aneuploidy is expected to be higher. This places highly diverged sex chromosomes at the bottom of plots in Fig. 3 and at the top of plots in Fig. 4. Taken together, we then predict a pattern where sexual antagonism is most likely the source of transitions to achiasmy in systems with young sex chromosomes and meiotic fidelity likely the driving force in meiotic transitions in clades with highly diverged sex chromosomes (Fig. 5).

The intuition behind our prediction in Fig. 5 is that sexual antagonism is more likely to drive achiasmy in young sex chromosomes, while aneuploidy avoidance dominates in older systems. This can be formalized by considering how these selective forces depend on the size of the recombining region (*L*). In our model, the total selective benefit of resolving sexual antagonism scales with


SA(L,s,h),

where SA is a function that increases with *L* being the number of loci, *s* being the strength of sexual antagonism, and *h* being the dominance factor. In contrast, the total selective benefit of avoiding aneuploidy through achiasmy is *k*(*L*), where *k* decreases with *L*, yielding an overall ratio of


$$\frac{k(L)}{SA(L,s,h)}.$$

Although the exact functional forms of *SA* and *k* are not currently known, biological reasoning suggests that they change in opposing directions as sex chromosomes diverge. As the PAR shrinks, the opportunity for sexually antagonistic variation is likely reduced (lowering SA), while the risk of aneuploidy is expected to increase as PAR shrinks; this is a pattern that is consistent with the fragile Y hypothesis. As a result, we expect this ratio to increase as *L* declines, shifting the balance of selection in favor of aneuploidy-driven transitions to achiasmy in older, more heteromorphic sex chromosomes. This analytic framework helps to explain the patterns observed in our heatmaps and motivates future work to better quantify how *SA* and *k* change across sex-chromosome evolution.

Looking ahead, several critical knowledge gaps emerge as essential areas for advancing our understanding of the evolution of achiasmatic meiosis. First, are transitions to achiasmy more common in lineages with highly diverged sex chromosomes or in lineages with largely homomorphic sex chromosomes? Brachycera (a Diptera suborder) is achiasmatic, while most other Diptera retain chiasmatic meiosis. Reconstructions of the sex-chromosome pair at the point of origin of Brachycera might reveal whether achiasmy evolved in a largely homomorphic sex-chromosome pair or a highly diverged sex-chromosome pair. A similar study would be challenging to replicate in Lepidoptera, a clade with female heterogamety and achiasmy. Unfortunately, the sister clade (Trichoptera) shares this female heterogametic system and also exhibits achiasmy (Blackmon et al. 2017). Finally, both clades typically exhibit highly diverged W chromosomes, but in some lineages, this ancestral W has even been lost (Goldsmith and Marec 2009; Fraïsse et al. 2017). This makes determining the state of the ZW sex chromosome at the point of achiasmy evolution difficult at best. Second, are small PAR sizes more likely to drive transitions to achiasmy in mammals? Rodent clades *Microtus* and *Gerbillinae* have multiple origins of achiasmatic sex chromosomes, offering the best opportunity to understand the relationship between achiasmy and sex-chromosome divergence in mammals (Wolf et al. 1988; Borodin et al. 2012). Estimating PAR size in closely related species that have retained chiasmatic segregation could help to reveal whether these recent transitions are associated with small or large PAR size. These data would clearly distinguish among the hypotheses explored in this paper.

## Supplementary Material

jkaf217_Supplementary_Data

## Data Availability

Data and analysis scripts are available in a GitHub repository: https://github.com/Andresdbp/achiasmy. Supplemental material available at G3 online.
