# Supplementary material for: Drivers of achiasmatic meiosis: sexual antagonism versus heteromorphy-dependent aneuploidy across sex-chromosome divergence
Source: G3 (Bethesda). 2025 Sep 19;15(11):jkaf217. doi: 10.1093/g3journal/jkaf217 (PMC12610934; doi:10.1093/g3journal/jkaf217)

1 Supplementary Material 1: Recursion Equations

In the recursion equations, $\overline{w_{♀}}$ and $\overline{w_{♂}}$ are used for brevity and are the mean fitness of females and males, respectively. Other variable identities are discussed in the main text of the paper.

1.1 Fitness Equations

$$\overline{w_{♀}} = X^{♀}_{fc}X^{♂}_{fc}w_1 + X^{♀}_{fc}X^{♂}_{fa}w_1 + X^{♀}_{fc}X^{♂}_{mc}w_2 + X^{♀}_{fc}X^{♂}_{ma}w_2 + X^{♀}_{fa}X^{♂}_{fc}w_1 + X^{♀}_{fa}X^{♂}_{fa}w_1 + X^{♀}_{fa}X^{♂}_{mc}w_2 + X^{♀}_{fa}X^{♂}_{ma}w_2 + X^{♀}_{mc}X^{♂}_{fc}w_2 + X^{♀}_{mc}X^{♂}_{fa}w_2 + X^{♀}_{mc}X^{♂}_{mc}w_3 + X^{♀}_{mc}X^{♂}_{ma}w_3 + X^{♀}_{ma}X^{♂}_{fc}w_2 + X^{♀}_{ma}X^{♂}_{fa}w_2 + X^{♀}_{ma}X^{♂}_{mc}w_3 + X^{♀}_{ma}X^{♂}_{ma}w_3$$

$$\overline{w_{♂}} = X^{♀}_{fc}Y^{♂}_{fc}w_4 + X^{♀}_{fc}Y^{♂}_{fa}w_7 + X^{♀}_{fc}Y^{♂}_{mc}w_5 + X^{♀}_{fc}Y^{♂}_{ma}w_8 + X^{♀}_{fa}Y^{♂}_{fc}w_7 + X^{♀}_{fa}Y^{♂}_{fa}w_7 + X^{♀}_{fa}Y^{♂}_{mc}w_8 + X^{♀}_{fa}Y^{♂}_{ma}w_8 + X^{♀}_{mc}Y^{♂}_{fc}w_5 + X^{♀}_{mc}Y^{♂}_{fa}w_8 + X^{♀}_{mc}Y^{♂}_{mc}w_6 + X^{♀}_{mc}Y^{♂}_{ma}w_9 + X^{♀}_{ma}Y^{♂}_{fc}w_8 + X^{♀}_{ma}Y^{♂}_{fa}w_8 + X^{♀}_{ma}Y^{♂}_{mc}w_9 + X^{♀}_{ma}Y^{♂}_{ma}w_9$$

1.2 Female Gamete Frequencies

$$X^{♀\prime}_{fc} = \frac{X^{♀}_{fc}X^{♂}_{fc}w_1 + .5X^{♀}_{fc}X^{♂}_{fa}w_1 + .5X^{♀}_{fc}X^{♂}_{mc}w_2 + .5(1-r_2)X^{♀}_{fc}X^{♂}_{ma}w_2 + .5X^{♀}_{fa}X^{♂}_{fc}w_1 + .5r_2X^{♀}_{fa}X^{♂}_{mc}w_2 + .5X^{♀}_{mc}X^{♂}_{fc}w_2 + .5r_2X^{♀}_{mc}X^{♂}_{fa}w_2 + .5(1-r_2)X^{♀}_{ma}X^{♂}_{fc}w_2}{\overline{w_{♀}}}$$

$$X^{♀\prime}_{fa} = \frac{.5X^{♀}_{fc}X^{♂}_{fa}w_1 + .5r_2X^{♀}_{fc}X^{♂}_{ma}w_2 + .5X^{♀}_{fa}X^{♂}_{fc}w_1 + X^{♀}_{fa}X^{♂}_{fa}w_1 + .5(1-r_2)X^{♀}_{fa}X^{♂}_{mc}w_2 + .5X^{♀}_{fa}X^{♂}_{ma}w_2 + .5(1-r_2)X^{♀}_{mc}X^{♂}_{fa}w_2 + .5r_2X^{♀}_{ma}X^{♂}_{fc}w_2 + .5X^{♀}_{ma}X^{♂}_{fa}w_2}{\overline{w_{♀}}}$$

$$X^{♀\prime}_{mc} = \frac{.5X^{♀}_{fc}X^{♂}_{mc}w_2 + .5r_2X^{♀}_{fc}X^{♂}_{ma}w_2 + .5(1-r_2)X^{♀}_{fa}X^{♂}_{mc}w_2 + .5X^{♀}_{mc}X^{♂}_{fc}w_2 + .5r_2X^{♀}_{mc}X^{♂}_{fa}w_2 + X^{♀}_{mc}X^{♂}_{mc}w_3 + .5X^{♀}_{mc}X^{♂}_{ma}w_3 + .5r_2X^{♀}_{ma}X^{♂}_{fc}w_2 + .5X^{♀}_{ma}X^{♂}_{mc}w_3}{\overline{w_{♀}}}$$

$$X^{♀\prime}_{ma} = \frac{.5X^{♀}_{fa}X^{♂}_{fc}w_1 + .5r_2X^{♀}_{fa}X^{♂}_{mc}w_2 + .5X^{♀}_{fa}X^{♂}_{ma}w_2 + .5r_2X^{♀}_{mc}X^{♂}_{fa}w_2 + .5X^{♀}_{mc}X^{♂}_{ma}w_3 + .5(1-r_2)X^{♀}_{ma}X^{♂}_{fc}w_2 + .5X^{♀}_{ma}X^{♂}_{fa}w_2 + .5X^{♀}_{ma}X^{♂}_{mc}w_3 + X^{♀}_{ma}X^{♂}_{ma}w_3}{\overline{w_{♀}}}$$

1.3 Male Gamete Frequencies (Sex-Linked)

$$X^{♂\prime}_{fc} = \frac{.5X^{♀}_{fc}Y^{♂}_{fc}w_4 + .5X^{♀}_{fc}Y^{♂}_{fa}w_7 + .5(1-r_1)X^{♀}_{fc}Y^{♂}_{mc}w_5 + .5X^{♀}_{fc}Y^{♂}_{ma}w_8 + .5r_1X^{♀}_{mc}Y^{♂}_{fc}w_5}{\overline{w_{♂}}}$$

$$X^{♂\prime}_{fa} = \frac{.5X^{♀}_{fa}Y^{♂}_{fc}w_7 + .5X^{♀}_{fa}Y^{♂}_{fa}w_7 + .5X^{♀}_{fa}Y^{♂}_{mc}w_8 + .5X^{♀}_{fa}Y^{♂}_{ma}w_8}{\overline{w_{♂}}}$$

$$X^{♂\prime}_{mc} = \frac{.5r_1X^{♀}_{fc}Y^{♂}_{mc}w_5 + .5(1-r_1)X^{♀}_{mc}Y^{♂}_{fc}w_5 + .5X^{♀}_{mc}Y^{♂}_{fa}w_8 + .5X^{♀}_{mc}Y^{♂}_{mc}w_6 + .5X^{♀}_{mc}Y^{♂}_{ma}w_9}{\overline{w_{♂}}}$$

$$X^{♂\prime}_{ma} = \frac{.5X^{♀}_{ma}Y^{♂}_{fc}w_8 + .5X^{♀}_{ma}Y^{♂}_{fa}w_8 + .5X^{♀}_{ma}Y^{♂}_{mc}w_9 + .5X^{♀}_{ma}Y^{♂}_{ma}w_9}{\overline{w_{♂}}}$$

$$Y^{♂\prime}_{fc} = \frac{.5X^{♀}_{fc}Y^{♂}_{fc}w_4 + .5r_1X^{♀}_{fc}Y^{♂}_{mc}w_5 + .5X^{♀}_{fa}Y^{♂}_{fc}w_7 + .5(1-r_1)X^{♀}_{mc}Y^{♂}_{fc}w_5 + .5X^{♀}_{ma}Y^{♂}_{fc}w_8}{\overline{w_{♂}}}$$

$$Y^{♂\prime}_{fa} = \frac{.5X^{♀}_{fc}Y^{♂}_{fa}w_7 + .5X^{♀}_{fa}Y^{♂}_{fa}w_7 + .5X^{♀}_{mc}Y^{♂}_{fa}w_8 + .5X^{♀}_{ma}Y^{♂}_{fa}w_8}{\overline{w_{♂}}}$$

$$Y^{♂\prime}_{mc} = \frac{.5(1-r_1)X^{♀}_{fc}Y^{♂}_{mc}w_5 + .5X^{♀}_{fa}Y^{♂}_{mc}w_8 + .5r_1X^{♀}_{mc}Y^{♂}_{fc}w_5 + .5X^{♀}_{mc}Y^{♂}_{mc}w_6 + .5X^{♀}_{ma}Y^{♂}_{mc}w_9}{\overline{w_{♂}}}$$

$$Y^{♂\prime}_{ma} = \frac{.5X^{♀}_{fc}Y^{♂}_{ma}w_8 + .5X^{♀}_{fa}Y^{♂}_{ma}w_8 + .5X^{♀}_{mc}Y^{♂}_{ma}w_9 + .5X^{♀}_{ma}Y^{♂}_{ma}w_9}{\overline{w_{♂}}}$$

1.4 Male Gamete Frequencies (Autosomal-Unlinked)

$$X^{\male\prime}_{fc} = \frac{.5X^{\female}_{fc}Y^{\male}_{fc}w_4+.25X^{\female}_{fc}Y^{\male}_{fa}w_7+.5(1-r_1)X^{\female}_{fc}Y^{\male}_{mc}w_5+.25X^{\female}_{fc}Y^{\male}_{ma}w_8+.25X^{\female}_{fa}Y^{\male}_{fc}w_7+.25X^{\female}_{fa}Y^{\male}_{mc}w_8+.5r_1X^{\female}_{mc}Y^{\male}_{fc}w_5}{\overline{w_{\male}}}$$

$$X^{\male\prime}_{fa} = \frac{.25X^{\female}_{fc}Y^{\male}_{fa}w_7+.25X^{\female}_{fc}Y^{\male}_{ma}w_8+.25X^{\female}_{fa}Y^{\male}_{fc}w_7+.5X^{\female}_{fa}Y^{\male}_{fa}w_7+.25X^{\female}_{fa}Y^{\male}_{mc}w_8+.5X^{\female}_{fa}Y^{\male}_{ma}w_8}{\overline{w_{\male}}}$$

$$X^{\male\prime}_{mc} = \frac{.5r_1X^{\female}_{fc}Y^{\male}_{mc}w_5+.5(1-r_1)X^{\female}_{mc}Y^{\male}_{fc}w_5+.25X^{\female}_{mc}Y^{\male}_{fa}w_8+.5X^{\female}_{mc}Y^{\male}_{mc}w_6+.25X^{\female}_{mc}Y^{\male}_{ma}w_9+.25X^{\female}_{ma}Y^{\male}_{fc}w_8+.25X^{\female}_{ma}Y^{\male}_{mc}w_9}{\overline{w_{\male}}}$$

$$X^{\male\prime}_{ma} = \frac{.25X^{\female}_{mc}Y^{\male}_{fa}w_7+.25X^{\female}_{mc}Y^{\male}_{ma}w_8+.25X^{\female}_{ma}Y^{\male}_{fc}w_7+.5X^{\female}_{ma}Y^{\male}_{fa}w_7+.25X^{\female}_{ma}Y^{\male}_{mc}w_8+.5X^{\female}_{ma}Y^{\male}_{ma}w_8}{\overline{w_{\male}}}$$

$$Y^{\male\prime}_{fc} = \frac{.5X^{\female}_{fc}Y^{\male}_{fc}w_4+.25X^{\female}_{fc}Y^{\male}_{fa}w_7+.5r_1X^{\female}_{fc}Y^{\male}_{mc}w_5+.25X^{\female}_{fa}Y^{\male}_{fc}w_7+.5(1-r_1)X^{\female}_{mc}Y^{\male}_{fc}w_5+.25X^{\female}_{mc}Y^{\male}_{fa}w_8+.25X^{\female}_{ma}Y^{\male}_{fc}w_8}{\overline{w_{\male}}}$$

$$Y^{\male\prime}_{fa} = \frac{.25X^{\female}_{fc}Y^{\male}_{fa}w_7+.25X^{\female}_{fa}Y^{\male}_{fc}w_7+.5X^{\female}_{fa}Y^{\male}_{fa}w_7+.25X^{\female}_{mc}Y^{\male}_{fa}w_8+.25X^{\female}_{ma}Y^{\male}_{fc}w_8+.5X^{\female}_{ma}Y^{\male}_{fa}w_8}{\overline{w_{\male}}}$$

$$Y^{\male\prime}_{mc} = \frac{.5(1-r_1)X^{\female}_{fc}Y^{\male}_{mc}w_5+.25X^{\female}_{fc}Y^{\male}_{ma}w_8+.25X^{\female}_{fa}Y^{\male}_{mc}w_8+.5r_1X^{\female}_{mc}Y^{\male}_{fc}w_5+.5X^{\female}_{mc}Y^{\male}_{mc}w_6+.25X^{\female}_{mc}Y^{\male}_{ma}w_9+.25X^{\female}_{ma}Y^{\male}_{mc}w_9}{\overline{w_{\male}}}$$

$$Y^{\male\prime}_{ma} = \frac{.25X^{\female}_{fc}Y^{\male}_{ma}w_8+.25X^{\female}_{fa}Y^{\male}_{mc}w_8+.5X^{\female}_{fa}Y^{\male}_{ma}w_8+.25X^{\female}_{mc}Y^{\male}_{ma}w_9+.25X^{\female}_{ma}Y^{\male}_{mc}w_9+.5X^{\female}_{ma}Y^{\male}_{ma}w_9}{\overline{w_{\male}}}$$

2 Supplementary Methods 1: GO Term Analysis

In this analysis, gene and Gene Ontology (GO) annotations were retrieved from the Ensembl BioMart database using R (version 4.2.2, packages biomaRt and topGO). The workflow began by establishing a connection to the Ensembl BioMart database, where we specified the dataset of interest. To ensure data quality and relevance, we filtered the gene list to include only a defined set of valid chromosomes, which consisted of numeric chromosomes (1–99) and common sex chromosome identifiers ("X", "Y", "W", "Z"). Gene annotations and GO term mappings were merged on the gene identifier to create a comprehensive dataset that linked each gene's chromosomal location with its GO terms. We then constructed a frequency table, where the counts of each GO term were tabulated per chromosome. This contingency-like table was organized such that each row corresponded to a GO term and each column to a chromosome, with cell values indicating the number of genes annotated with a particular GO term on a given chromosome.

For the enrichment analysis, we adopted Fisher's exact test to evaluate whether specific GO terms were overrepresented on a chromosome of interest compared to other chromosomes. A helper function was defined to construct a 2×2 contingency table for each GO term by calculating the number of genes with and without the term on both the target chromosome and all other chromosomes. Fisher's test was used on these contingency tables, with the test set to one-tailed ("greater"), to generate a p-value for the enrichment of each GO term. After calculating p-values for all GO terms, a False Discovery Rate (FDR) correction was applied to account for multiple testing, and the results were compiled into a data frame that was sorted by the adjusted p-value.

The entire pipeline was applied to Drosophila melanogaster and Homo sapiens by switching the Ensembl dataset accordingly, thereby enabling cross-species comparisons of GO term enrichment on the Y chromosome.

3 Supplementary Figure 1: Equilibrium Time Under Sexual Antagonism

Time to equilibrium for achiasmatic mutations on different genomic locations under sexual antagonism.

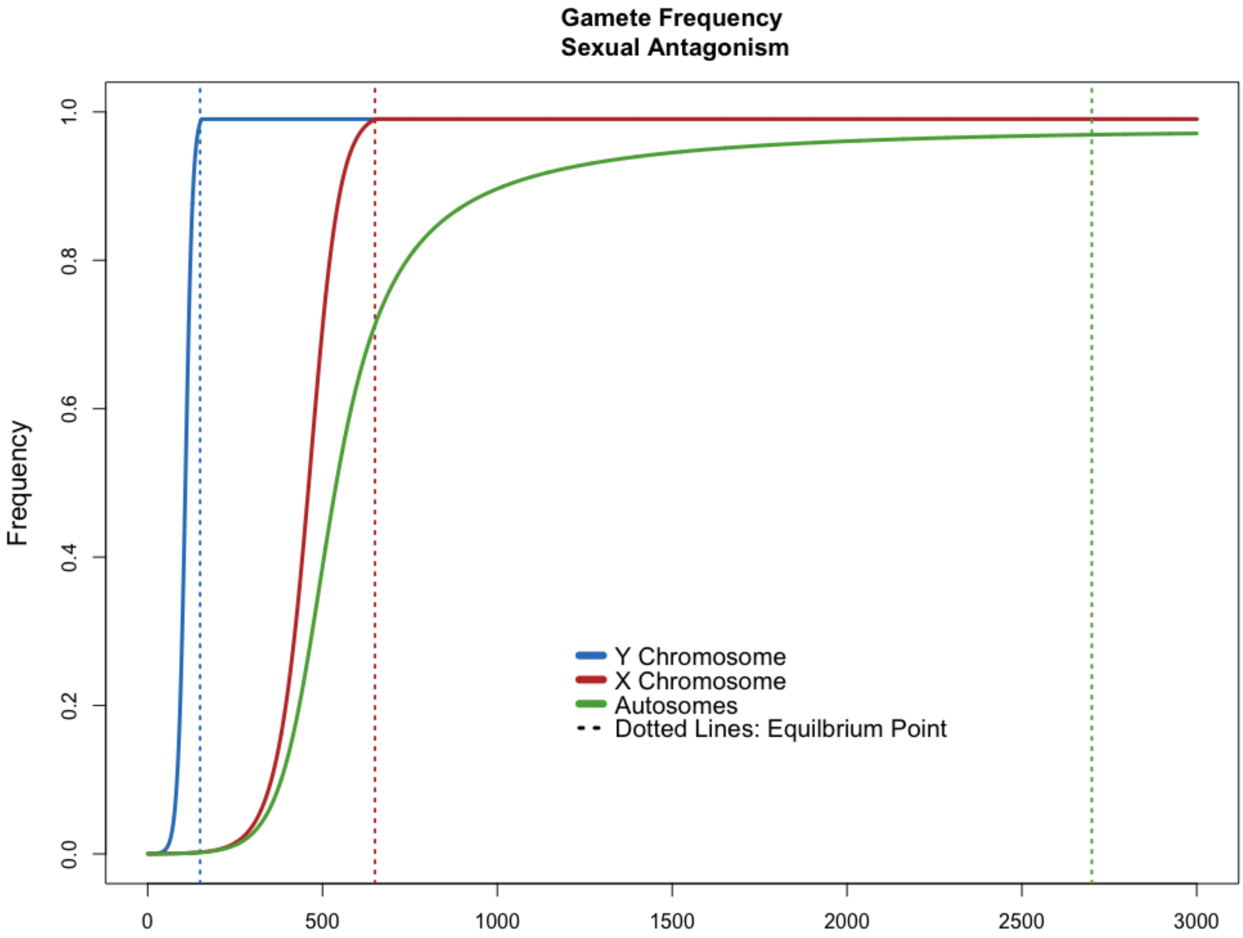

4 Supplementary Figure 2: Equilibrium Time Under Heteromorphy-dependent Aneuploidy

Time to equilibrium for achiasmatic mutations on different genomic locations under heteromorphy-dependent aneuploidy.

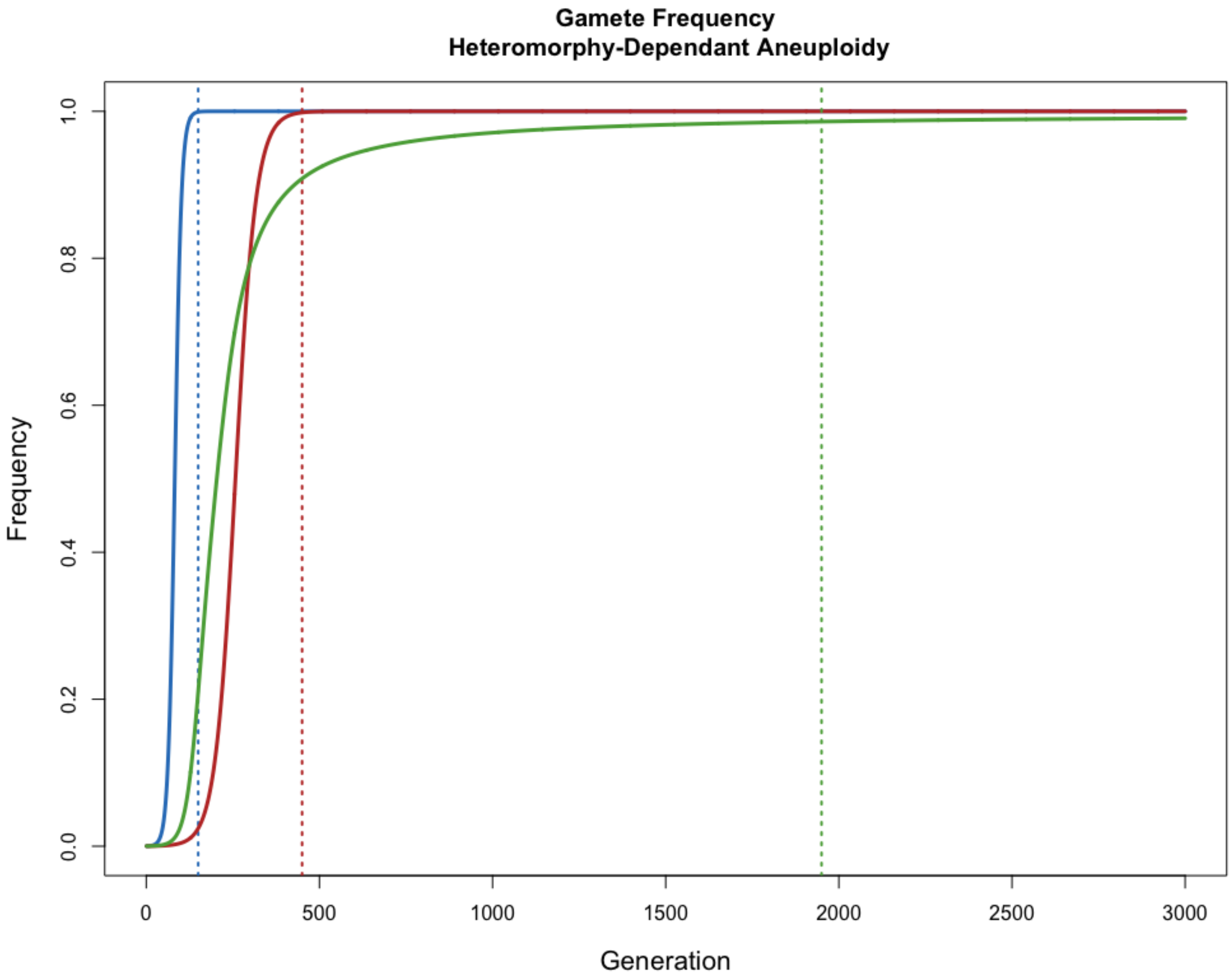

5 Supplementary Figure 3: Effect of Dominance Factor for Sexual Antagonism on Fixation

Effect of dominance factor h = *0.5* on the fixation of the achiasmy mutation across genomic regions

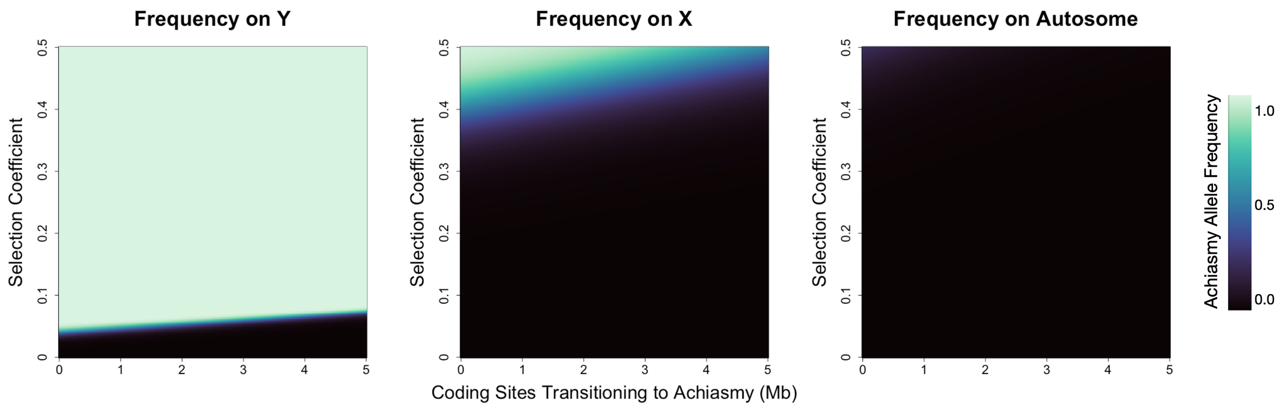

6 Supplementary Figure 4: Effect of Dominance Factor for Sexual Antagonism on Fixation

Effect of dominance factor h = *0* on the fixation of the achiasmy mutation across genomic regions

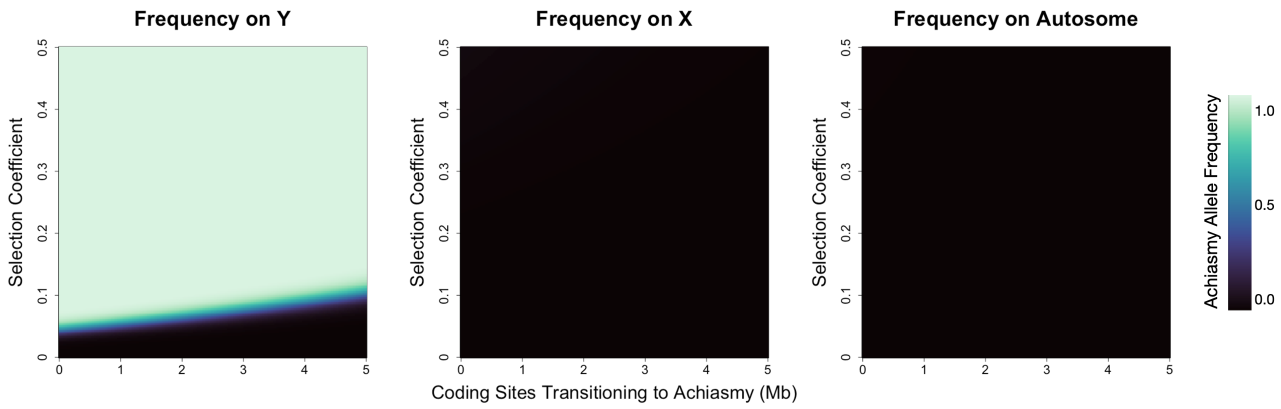

7 Supplementary Figure 5: Effect of Recombination Distance R2 on Fixation under Sexual Antagonism

Effect of higher recombination distance for $R2 = 0.3$ on the fixation of the achiasmy mutation across genomic regions

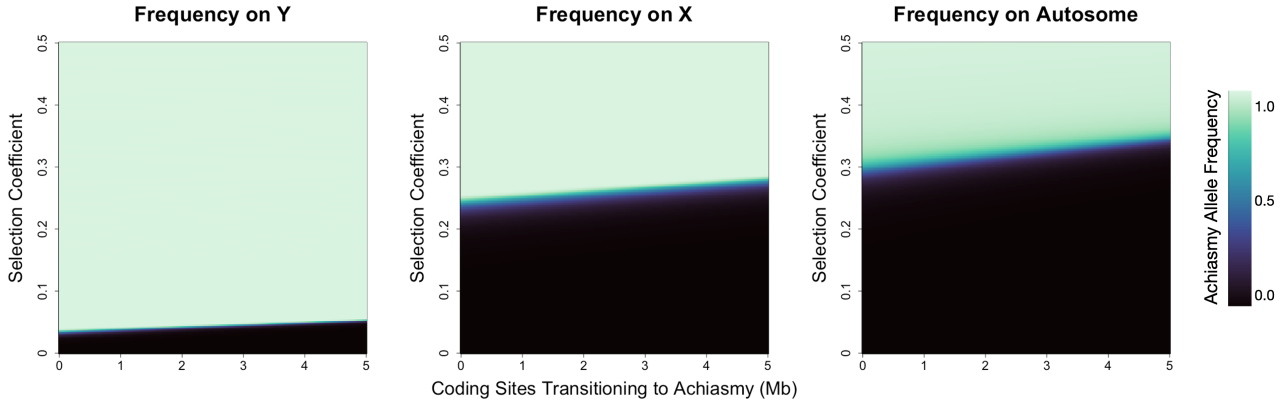

Supplement: jkaf217_Supplementary_Data [file jkaf217_supplementary_data.pdf]
